# Object-based Encoding in Visual Working Memory: Evidence from Memory-driven Attentional Capture

**DOI:** 10.1038/srep22822

**Published:** 2016-03-09

**Authors:** Zaifeng Gao, Shixian Yu, Chengfeng Zhu, Rende Shui, Xuchu Weng, Peng Li, Mowei Shen

**Affiliations:** 1 Department of Psychology, Zhejiang University, Hangzhou, China; 2 Department of Psychology, Hangzhou Normal University, Hangzhou, China; 3 School of Education & Management, Yunnan Normal University, Kunming, China

## Abstract

Visual working memory (VWM) adopts a specific manner of object-based encoding (OBE) to extract perceptual information: Whenever one feature-dimension is selected for entry into VWM, the others are also extracted. Currently most studies revealing OBE probed an ‘irrelevant-change distracting effect’, where changes of irrelevant-features dramatically affected the performance of the target feature. However, the existence of irrelevant-feature change may affect participants’ processing manner, leading to a false-positive result. The current study conducted a strict examination of OBE in VWM, by probing whether irrelevant-features guided the deployment of attention in visual search. The participants memorized an object’s colour yet ignored shape and concurrently performed a visual-search task. They searched for a target line among distractor lines, each embedded within a different object. One object in the search display could match the shape, colour, or both dimensions of the memory item, but this object never contained the target line. Relative to a neutral baseline, where there was no match between the memory and search displays, search time was significantly prolonged in all match conditions, regardless of whether the memory item was displayed for 100 or 1000 ms. These results suggest that task-irrelevant shape was extracted into VWM, supporting OBE in VWM.

Visual working memory (VWM) temporarily holds and manipulates a limited set of visual information (e.g. 3–4 simple visual objects at most) for the ongoing task (see refs 1 and 2 for reviews; but see ref. 3 for a different view). Although the capacity of VWM is rather limited, it has been suggested that VWM extracts perceptual information from a scene via an uneconomical *object-based encoding* (OBE) manner, which may be detrimental to the ongoing task^4^,^5^,^6^,^7^,^8^. Particularly, even if only one feature dimension is selected for entry into VWM, the other task-irrelevant feature dimensions, which together with the target dimension belong to the same object, are also extracted into VWM.

The establishment of OBE in VWM is important, not only because it enables us to understand the encoding mechanisms of VWM, but also because it sheds critical light on the intimate interaction between visual perception, VWM, and visual attention^7^,^8^. However, empirical evidence supporting OBE is controversial. Earlier studies of VWM assumed that OBE took place in VWM, by showing that irrelevant spatial information was encoded into VWM automatically^9^,^10^. Later studies revealed that participants actually could restrict their selection to the task-relevant dimension while filtering out the irrelevant ones^11^,^12^, at least at a high memory load condition (e.g., remembering 6 objects^13^).

However, recent studies provided consistent evidence suggesting that OBE robustly exists in VWM when probing an *irrelevant-change distracting effect*^4^,^5^,^6^,^7^,^8^,^14^,^15^,^16^,^17^,^18^. This distracting effect is revealed in a change-detection task, wherein the participants are required to remember one feature dimension of multi-featured objects and ignore the other one(s). Critically, the type of change of the irrelevant dimension (no irrelevant change vs. irrelevant change) is manipulated. For example, participants are presented with two coloured shapes and required to remember shape while ignoring colour. After an interval, a test array is presented, and participants judge whether a change in the target dimension takes place relative to the memorized item. Critically, the task-irrelevant dimension can also change (e.g. 50% of trials), by adopting a new feature that has not appeared in the memory array or an old feature belonging to another old object. A significant distracting effect is consistently observed by demonstrating that the change of the irrelevant feature considerably impairs the change-detection performance of the target dimension^4^,^5^,^6^,^7^,^8^,^14^,^15^,^16^,^17^,^18^. Moreover, congruent with the behavioural evidence, ERP studies showed that the irrelevant-feature change elicited a more negative ERP component of the anterior N2 relative to the no-change condition^8^,^16^,^17^,^18^. These results suggest that the irrelevant features are automatically selected into VWM in an object-based manner. Moreover, this OBE has been consistently revealed in both low (e.g. 2 objects) and high (e.g. 6 or 8 objects) memory load conditions^4^,^5^,^13^, in various feature dimensions (e.g. colours, shapes, orientations, sizes)^5^,^6^,^8^,^14^,^15^,^17^, and with different encoding times (e.g. 100 ms, 1000 ms)^5^; it also remains constant throughout age from 6 to 72 years^19^. Moreover, the maintenance of irrelevant information in VWM can last for at least 1000 ms after the offset of the memory array^20^ and is not modulated by the probability of the irrelevant-feature change, exhibiting the distracting effect with an irrelevant-change probability of 16%, 20%, and 50%.^5^

While at the current stage OBE has received strong support by the irrelevant-change distracting effect, the results may be confounded by the special settings employed in the task. For instance, it has been argued that because the VWM task probing the irrelevant-change distracting effect ‘*required change detection, and the marker of obligatory encoding of irrelevant features also involved a change, it remains to be seen whether such strong evidence of OBE depends on task demands*’ (cf. To OBE or not to OBE? *News from the field*, Attention, Perception & Psychophysics, 2013, 75, 632–633). Therefore, it is possible that the OBE revealed by the irrelevant-change distracting effect reflects a processing strategy instead of an essential VWM processing characteristic. That is, although the participants were informed to ignore the irrelevant feature, they noticed the change of the irrelevant feature during the practice or formal experiment and deduced that it could be task-relevant and therefore changed their processing strategy. Supporting this possibility, Chen and Wyble^21^ recently revealed that once the participants noticed the test content, they changed their processing strategy immediately. Therefore, an Achilles’ heel may be hidden in previous studies employing the irrelevant-change distracting effect, which represents a severe challenge to the established OBE in VWM.

To this end, the current study conducted a strict examination of the OBE in a new paradigm while keeping the irrelevant dimension stable during the experiment. We achieved this by examining whether the task-irrelevant dimension of multi-featured objects could lead to memory-driven attentional capture. Accumulating evidence has consistently demonstrated that the contents of VWM can automatically guide attention^22^,^23^,^24^,^25^,^26^, although it is also subjected to some degree of cognitive control^27^,^28^,^29^,^30^,^31^. In these studies, a simple object (e.g. a square) was usually presented at the beginning of each trial and participants had to retain this object until the end. During the VWM maintenance interval, a memory-irrelevant visual search array appeared, containing a tilted target bar among vertical distractor bars. Each bar was embedded in a shape (e.g. circle), one of which could match the object in VWM. Even if the memory-matching object was completely unrelated to the location of the search target, search performance (e.g. search time) was significantly impaired when the distractor bars were embedded in the memory-matching items, suggesting that attention was automatically deployed towards items that matched the contents of VWM.

We assume that if OBE indeed takes place in VWM, then the task-irrelevant feature should also induce memory-driven attentional capture. Soto and Humphreys^23^ seemed to support this prediction, although they aimed at exploring the boundary conditions of memory-driven attentional capture. Specifically, using the aforementioned method, they required the participants to memorize the shape of a coloured object while ignoring its colour and manipulated the identity type of the memory-matching item, which always enclosed a distractor bar. There were four matching types: One of the objects in the search display could match the shape (relevant-match), the colour (irrelevant-match) or both dimensions (conjunction match) of the memorized object, or did not match any dimension (neutral). Additionally, the authors also manipulated the probe type, consisting of a coloured probe, in which the probe colour could change in 50% of the trials, and a black probe, in which the probe colour always changed to the unused colour black. They found that the conjunction-match significantly slowed down the search time relative to the other match conditions, and the irrelevant-match under the black-probe condition considerably slowed down the search time relative to the neutral baseline. Critically, there were more incorrect first fixations on the irrelevant-match and conjunction-match conditions than the relevant-match condition regardless of the probe type. These results, therefore, imply that the irrelevant colour was encoded into VWM and hence guided the deployment of attention in visual search.

Although Soto and Humphreys^23^ provided encouraging evidence, caution should be exerted in concluding that OBE indeed takes place for the following two reasons: First, the typical marker of memory-driven attention capture, to some extent, is lacking in the study of Soto and Humphreys^23^: There was no clear evidence that the relevant-match captured attention. However, both behavioural and eye-movement data showed that that the irrelevant colour was more effective in capturing attention, which could potentially explain the results on the conjunction-match. Consequently, the attention capture may be due to the special nature of colour in capturing attention (see also ref. 32) and cannot be generalized to other feature dimensions. Corroborating this possibility, a study implied that the stimulus attributes of memorized objects affect memory-driven attention capture^33^. Second, the irrelevant colour changed between the memorized item and probe (50% in the colour-probe condition and 100% in the black-probe condition), which, as mentioned for the irrelevant-change distracting effect, may potentially affect participants’ processing strategy. Indeed, Olivers *et al*.^34^ adopted a similar design as Soto and Humphreys^23^, keeping the irrelevant dimension constant between the memorized item and probe, and revealed a reversed profile: The memory-driven attention capture took place only under the relevant-match condition but not under the irrelevant-match condition, regardless of whether the irrelevant dimension was shape or colour. Sala and Courtney^35^ further replicated this finding by using location and texture as the tested dimension. However, we also noticed that there was a 4-second and 1–3-second blank interval between the memorized item and search display in Olivers *et al*.^34^ and Sala and Courtney^35^, respectively. Therefore, the task-irrelevant dimension might have decayed by then, considering that Logie *et al*.^20^ suggested that the task-irrelevant feature began to decay roughly at 1 or 1.5 seconds. Overall, it is of necessity to re-examine whether the task-irrelevant feature of a memorized object could indeed lead to memory-driven attention capture.

We addressed this issue by using a paradigm similar to Soto and Humphreys^23^, since Soto *et al*. had already provided kind of weak yet useful information on OBE and that the time parameter between memory item and search display (188 ms) did not lead to significant decay for the potentially encoded information. However, there were four key differences between the current and Soto *et al*.’s study^23^. First, we set colour as the task-relevant dimension while shape was irrelevant. Second, in line with Olivers *et al*.^34^, we kept the irrelevant dimension constant between the memorized item and probe. The two settings provide a complimentary test of Soto and Humphreys^23^ while avoiding the potential caveats, forming a strict examination of OBE in VWM and enabling us to explore whether the OBE revealed in Soto and Humphreys^23^ was limited to colour. Moreover, since Soto *et al*. did not provide clear evidence of attention capture when shape was task-relevant, we considered that if attention capture were revealed for the irrelevant shape, we could provide even stronger evidence supporting OBE. Third, instead of informing the participants about all the possible match conditions as well as their probabilities, we told the participants that the visual search task was irrelevant to the memory task. We adopted this instruction to prevent the participants from strategically searching the match item in the search display to refresh the stored representation. Fourth, we examined the potential influence of top-down control over OBE by manipulating the exposure time of the memory array (100 ms vs. 1000 ms). Sander *et al*.^36^,^37^ suggested that the exposure time of the memory array could modulate top-down control: At a short exposure (e.g. 100 ms), low-level feature binding takes place rapidly to integrate fleeting perceptual information into coherent representations stored in VWM; then, top-down control, supported by the prefrontal cortex, begins to interact with the already stored representation and acts over a longer duration than low-level feature binding. Consequently, a short exposure time is sufficient for feature binding but not for top-down control. The OBE may reflect the final result of low-level feature binding. Shen *et al*.^5^ examined this possibility by using two exposure times (100 ms vs. 1000 ms) and found that exposure time did not modulate the irrelevant-change distracting effect. Considering that at a longer exposure time, the top-down control may inhibit the task-irrelevant dimension and hence change the search result pattern – for instance, Sawaki and Luck^38^ found active suppression of the task-irrelevant dimension with an exposure time of 400 ms – we hence further examined the effect of exposure time on memory-driven attention capture. Additionally, to our knowledge, no study has explored this exposure-time issue for attention capture driven by the task-irrelevant dimension in VWM. We aimed at closing this gap.

## Methods

### Participants

Twenty-two volunteers (10 females; *M* = 19.8 ± 1.8 years old) from Zhejiang University participated in the experiment for payment or course credit. All participants provided signed informed consent and had normal color vision and normal or corrected-to-normal visual acuity. The study was approved by the Research Ethics Board of Zhejiang University, and was performed in accordance with the approved guidelines.

### Stimuli and apparatus

The experiment was programmed via Matlab (The MathWorks, Natick, MA) with Psychophysics Toolbox^39^. The stimuli were displayed on a SAMSUNG SynchMaster 997MB color monitor with a resolution of 1024 × 768 pixels at a 100-Hz refresh rate. A viewing distance of 90 cm was maintained by a chin rest.

The memory cue was a colored shape, with color as the target feature. Similar to Soto and Humphreys^23^, the color was randomly selected from the following five values: red (255, 0, 0; in RGB), green (0, 128, 0), blue (0, 0, 255), yellow (255, 255, 0) and pink (255, 192, 203). The shape could be a circle (1.27° × 1.27° of visual angle), a square (1.15° × 1.15°), a star (1.59° × 1.59°), a triangle (1.46° × 1.08°), and a hexagon (1.59° × 1.15°). It is of note that we replaced the diamond in Soto and Humphreys^23^ with a star, to avoid the potential confusion between square and diamond.

The three distractor lines were vertical, whereas the target was tilted 12° either to the left or to the right. The length of the lines was 0.36° of visual angle and their width was 0.08° of visual angle.

### Procedure

**Figure 1:**
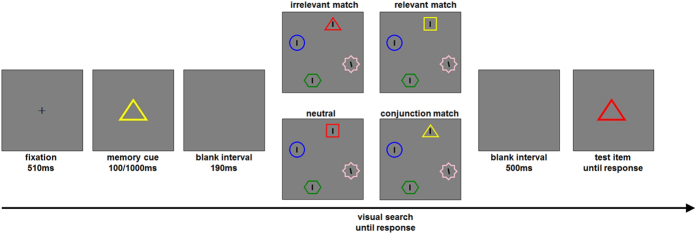
Illustration of a trial used in the experiment. Participants first viewed the memory cue for 100 ms or 1000 ms and retained its color. In the retention interval, they searched for a target line and specified its direction. Finally, they were shown a second item and judged whether its color matched the memory cue (here the color changed).

After a 510-ms fixation, a memory item appeared at the centre for 100 or 1000 ms, and participants were instructed to memorize its colour (see Fig. 1). After a blank interval of 190 ms, a search display, in which four black lines embedded within four distinct coloured shapes, was presented. The stimuli were evenly distributed over an imaginary circle (with a radius of 4.46° of visual angle). Participants were required to find the tilted line as quickly and accurately as possible and indicate whether the line tilted to the left or right. Finally, after a 500-ms blank interval, a test item appeared and the participants made an un-speeded judgement as to whether the colour matched the memory item (emphasizing accuracy). The shape of the test item was always the same as the memory item.

We adopted a 2 (Exposure Time: 100 ms vs. 1000 ms) × 4 (Memory-search Match: irrelevant match, relevant match, conjunction match, and neutral) within-subjects design. The four memory-search match conditions were determined by how the memory item matched one of the stimuli enclosing a *distractor* in the search display. In particular, in the irrelevant-match condition, one stimulus in the search display shared the shape (but not colour) of the memory cue; in the relevant-match condition, one stimulus in the search display shared the colour (but not shape) of the memory cue; in the conjunction-match condition, the memory cue was displayed as an item in the search display; and in in neutral condition, no feature of the memory cue appeared in the search display. In all conditions, the other stimuli in the search display did not contain any colour or shape value from the memory cue. Critically, the matching stimulus in the search display was always invalid across trials. In line with Soto and Humphreys^23^, 60% of the trials were invalid (i.e. irrelevant-match, relevant-match, and conjunction-match condition each consisted of 20% of the trials), and the remaining 40% were neutral. The four match conditions were displayed randomly. The participants were informed that the visual search task was irrelevant to the memory task.

The whole experiment was divided into two blocks according to the exposure time of the memory cue, with the order being counterbalanced among participants. Each block contained 140 trials, with 56 trials for the neutral condition and 84 trials for the three match conditions (i.e. 28 trials for each invalid condition). Before each block, participants first finished 25 practice trials before entering into the formal experiment. Every 35 trials, the participants had a 3–5-minute rest. The whole experiment lasted about 40 minutes.

### Analysis

Two-way repeated measures Analysis of Variance (ANOVA) with Exposure Time and Memory-search Match as within-subject factors were conducted on both the accuracy and search time of the search task, and the accuracy of the memory task. Significant main effect of Memory-search Match was followed by post-hoc contrasts, with the *p-*value being Bonferroni-corrected.

For the accuracy of the search task, only trials with correct responses in the memory task were analyzed. For the search times, only trials with correct responses in both the memory and the search tasks were analyzed; search times shorter than 200 ms or longer than 2000 ms were removed, resulting in 92.4% of the trials into analysis.

## Results

The accuracy for VWM and search tasks was summarized in Table 1. The overall accuracy of the memory task was 95% (SD = 0.026).

The two-way ANOVA revealed that the memory accuracy was slightly higher in the 1000-ms condition (*M* = 96.4%) than in the 100-ms condition (*M* = 94.5%), *F* (1, 21) = 16.08, *p* = 0.001, η_p_^2^ = 0.43. The other effects were not significant, *p*s > 0.05. The overall accuracy of the search task reached ceiling, with an average accuracy of 99% (SD = 0.015). The two-way ANOVA did not reveal any significant effect, *p*s > 0.05.

**Figure 2:**
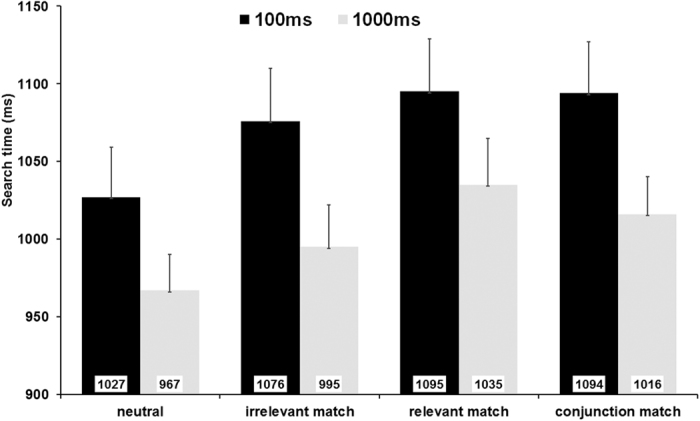
Visual search times in four match conditions. Error bar represents standard error.

For the search time of the visual search task (see Fig. 2), the two-way ANOVA yielded a significant main effect of Exposure Time, *F* (1, 21) = 14.68, *p* = 0.001, η_p_^2^ = 0.41, showing that the search time was significantly longer in the 100-ms condition (*M* = 1074 ms) than in the 1000-ms condition (*M* = 1003 ms). A significant main effect of Memory-search Match was revealed, *F* (3, 63) = 14.38, *p* < 0.001, η_p_^2^ = 0.41. Post-hoc contrasts revealed that the search time was significantly faster in the neutral condition (*M* = 998 ms) than in both the relevant-match (*M* = 1065 ms; *p* < 0.001) and conjunction-match condition (*M* = 1055 ms; *p* = 0.001). Critically, we found that the search time was also significantly faster in the neutral condition than in the irrelevant-match condition (*M* = 1036 ms; *p* = 0.001). Additionally, we found that the search time was faster in the irrelevant-match than relevant-match condition (*p* = 0.036), yet no difference was found between irrelevant-match and conjunction-match (*p* = 0.799) or between relevant-match and conjunction-match (*p* = 1.0).

The interaction between Exposure Time and Memory-search Match did not reach significance, *F* (3, 63) = 0.65, *p* = 0.59, η_p_^2^ = 0.03, suggesting that a similar automatic guidance due to the stored VWM content took place between 100 ms and 1000 ms.

## Discussion

The current study aimed at re-examining object-based encoding (OBE) in VWM, as the predominant evidence supporting OBE came from an irrelevant-change distracting effect, which might have been affected by participants’ processing strategy since both the relevant feature and irrelevant feature could change. We overcame the potential change confound by taking advantage of a phenomenon named memory-driven attention capture. We inserted a visual search task 190 ms after the offset of the memory array and explored whether attention during visual search was automatically deployed toward the item that matched the task-irrelevant feature of the memorized object. In line with previous studies^22^,^23^,^24^,^25^,^26^, we found that both relevant-match and conjunction-match significantly slowed down the search time relative to the neutral baseline, suggesting that the task-relevant feature in VWM could automatically capture attention during the visual search. Moreover, a similar result profile was revealed in the irrelevant-match condition, suggesting that the irrelevant feature of the memory cue was also stored in VWM and influenced the attention deployment. Therefore, the current study provides new evidence supporting OBE in VWM.

The current study adds to the VWM literature in several ways. First, it replicated and extended Soto and Humphreys’ study^23^, and thus was the second to confirm that the task-irrelevant feature of the memorized item could lead to memory-driven attention capture. Moreover, different from the finding of Soto and Humphreys^23^ that attention capture occurred predominately in the irrelevant-match condition, we demonstrated the attention capture in both relevant-match and irrelevant-match condition. Since the tested stimuli were roughly the same while the probe was displayed in distinct ways between the two studies, these results hence provide evidence supporting our speculation that the presentation of the probe might have influenced the participants’ processing manner in Soto and Humphreys^23^, making the task-irrelevant dimension more obvious to the participants.

Second, the current automatic attention deployment was driven by irrelevant shape instead of colour, suggesting that colour is not unique in capturing attention as an irrelevant dimension. Therefore, Soto and Humphreys^23^ and the current study together provide consistent evidence that OBE takes place in VWM and leads to attention capture. Additionally, both studies support the view that the absence of attention capture in Olivers *et al*.^34^ and Sala and Courtney^35^ is related to the long blank interval after the offset of the memory array. Future studies tapping OBE should pay attention to the survival time of the irrelevant feature in VWM.

Third, the current study for the first time examined the influence of top-down control on attention capture driven by the task-irrelevant dimension, by manipulating the exposure time of the memory array. Using the same time parameters, Sander *et al*.^37^ demonstrated that the distracting effect, which was caused by distractors appearing at distinct locations from the target, was reduced as the exposure time increased from 100 to 1000 ms. The distracting effect even vanished in young adults at 1000 ms. However, we found that although the distracting effect showed a decreasing trend (from 45 ms in the 100-ms condition to 28 ms in the 1000-ms condition), there was no statistical difference between the two (*t*(21) = 1.28, *p* = 0.21, Cohen’ *d* = 0.399). This finding, on the one hand, is congruent with Shen *et al*.^5^, suggesting that the top-down control cannot erase the OBE; on the other hand, it provides additional evidence implying that there are distinct mechanisms between feature-based filtering (e.g. the current study) and location-based filtering^37^. Additionally, we have to point out that the current study focused on whether the OBE could be erased by top-down control instead of whether the attention capture could vanish via top-down control. Therefore, we did not manipulate the availability of top-down control during the visual search task, as elegantly conducted in the study by Han and Kim^28^, in which they found that the time course of cognitive control was a critical factor in determining when VWM content affected attention.

Fourth, the prolonged search time under the irrelevant-match condition suggests that the irrelevant feature extracted by OBE is in an active state in VWM. This could partially explain the newly revealed fact that the task-irrelevant feature significantly reduced the precision of relevant features stored in VWM^40^. Meanwhile, we also noticed that Sawaki and Luck^38^ revealed evidence for suppressing the task-irrelevant feature. However, we argue that the two findings are not necessarily contrary to each other. Particularly, it has been suggested that the memory-driven attention capture is also modulated by cognitive control^27^,^30^,^31^, which needs time to take place^28^,^29^. The blank interval between memory cue and visual display was at least 510 ms shorter in the current study than in the study of Sawaki and Luck. Therefore, cognitive control was quite possibly not implemented in our study but was in Sawaki and Luck^38^. Combining the current study and Sawaki and Luck^38^ and Logie *et al*.^20^, it seems that all the highly discriminable information is initially encoded into VWM via an OBE manner; however, the task-irrelevant feature is gradually suppressed within VWM and finally decays (see also ref. 41).

Finally, the current study revealed that relevant-match condition had a longer search time than irrelevant-match condition. There are two possible explanations. First, the irrelevant feature is at an inferior status compared to the relevant feature in VWM. Second, the physical attributes may influence the capability of capturing attention^23^,^33^,^42^. The used colours may be more effective in capturing attention than the shapes. Future studies are required to figure out the reasons.

## Additional Information

**How to cite this article**: Gao, Z. *et al*. Object-based Encoding in Visual Working Memory: Evidence from Memory-driven Attentional Capture. *Sci. Rep.* **6**, 22822; doi: 10.1038/srep22822 (2016).

## Figures and Tables

**Table 1:** The averaged accuracy in the four memory-search match conditions for both visual search and VWM tasks.

		Memory-search Match			
	Exposure time	Neutral	Irrelevant match	Relevant match	Conjunction match
Visual search	100 ms	98%	98%	98%	99%
	1000 ms	99%	99%	99%	99%
VWM	100 ms	94%	94%	94%	96%
	1000 ms	97%	97%	96%	96%
